# Event-related potentials and the study of memory retrieval: A critical review

**DOI:** 10.1590/S1980-57642009DN20400003

**Published:** 2008

**Authors:** Antonio Jaeger, Maria Alice de Mattos Pimenta Parente

**Affiliations:** 1 Pós-graduação em Psicologia, Instituto de Psicologia, Universidade Federal do Rio Grande do Sul, Porto Alegre, Brazil.

**Keywords:** memory, retrieval, event-related potentials

## Abstract

Memory retrieval has been extensively investigated by a variety of techniques and methodological approaches. The present article reports a critical review on the research investigating this subject by means of event-related potentials (ERP). The main goal is to elucidate the key contributions of this technique regarding episodic memory retrieval, as well as to perform a critical analysis taking into account its major advantages and limitations in the framework of current cognitive neuroscience. Considerations concerning its theoretical contributions and implementation in national universities are also discussed.

## Abstract (pt)

A recuperação de memórias tem sido extensivamente pesquisada por uma variedade de técnicas e abordagens metodológicas. No presente artigo, é conduzida uma revisão crítica das pesquisas que investigaram este tema através da utilização da técnica denominada potenciais relacionados ao evento (PRE). O objetivo principal é elucidar as principais contribuições proporcionadas por esta técnica referente aos processos de recuperação de memórias episódicas, assim como realizar uma análise crítica levando em consideração as principais vantagens e limitações desta abordagem dentro do contexto atual da neurociência cognitiva. Um balanço de suas contribuições teóricas e de sua utilização em universidades nacionais é por fim desenvolvido.

The study of memory retrieval has undergone dramatic change over the last few decades.^1^

A significant improvement in memory retrieval research was made possible by the development of techniques that enabled scientists to monitor, directly and indirectly, brain activity during the process of retrieval. In contrast to neuroimaging techniques such as Functional Magnetic Resonance Imaging (fMRI) and Positron Emission Tomography (PET), which provide spatially accurate images of brain activities during periods of 2 to 3 seconds, Event-related potentials (ERP) provides “real time” monitoring of electrical activities of the brain.^2^

Therefore, the present article aimed to demonstrate how ERPs are elucidating essential assumptions related to the process of memory retrieval. A first discussion outlines the specific advantages and limitations of ERP over other cognitive neuroscience procedures. Subsequently, the particularities and specificities of memory retrieval will be focused on to achieve the main objective of this study: How can results in ERP studies on memory retrieval be interrelated to particular methodological variables (such as type of stimuli and timing)? And how, taking into account these methodological aspects, can ERP contribute to theoretical debates on different processing and neural bases involved in memory retrieval?

Following this analytic review, a broader critical analysis on these ERP contributions and potential is included in the critical analysis section, and considering the massive attention that has been dedicated to fMRI by the scientific community, some considerations on the specificities of both techniques are subsequently included.

In order to address the aforementioned questions and to properly analyze the data, a broad search in the literature was performed between January and July 2008. The following online databases were consulted during this period: Pubmed, Medline, Psychinfo and Scielo. The key words employed in this search were the combinations between Retrieval, Memory, ERP, Event-related potentials, and Electrophysiological. All research reports found on ERP and retrieval memory were included. There were no constraints concerning the year of publication.

## ERP

The electroencephalogram (EEG) consists basically of electrical fields recorded continuously over the human scalp. These electrical fields result in oscillatory waveforms which are largely employed in neurology as a technique for the diagnosis of neurological disorders.^3^ Although ERP employs the same brain electrical fields as the EEG, the former records electrical fields resulting from discrete events, that is, the electrical fields generated by brain activity associated to the presentation of specific experimental stimuli.^4^ Therefore, each stimulus presentation elicits a specific waveform. After being averaged, the waveforms belonging to the stimuli classes of interest (for example, emotional vs. neutral stimuli) are analyzed considering their voltage differences.

The differences in voltage generated by each distinct group of stimuli indicate that different electrophysiological brain activities were elicited by each class of stimuli.^2^ The voltage differences may be observed over time (milliseconds) from the moment each stimulus is presented. The data analysis normally includes the time-course of these differences as well as their topographical distribution over the scalp.^5^

The ERP signal is generated by the synchronized and cooperative activity of a large-scale neuronal network, on a time scale similar to that of single-neuron activity.^4^ These electrical fields are generated mainly over the dendrites, and are resultant of the sum of extra cellular fields generated by the post-synaptic activity of the cortical pyramidal cells.^6^ These currents flow through the cell membrane and over the intra and extra cellular spaces, and when a large group of neighboring neurons are simultaneously activated, the field potentials may be recorded over the scalp, yielding the ERP signal.^4^ In order to generate a field potential strong enough to be recorded over the human scalp, the neurons have to be distributed in a particular geometric configuration, that is, they should be distributed in such a way that their individual potential fields can be summed. This generates a ‘dipole moment’, which consists of a current movement provoked by positively and negatively charged fields. This geometric arrangement of neurons is called ‘open field’ and constitutes a large number of neurons distributed in parallel, as typically occurs with cortical pyramidal cells.^3,7^

Therefore, one of the main limitations of the ERP technique is that only sufficiently strong potentials generated synchronically by open fields are recordable across the scalp. Certain important electrical activities are probably not recorded in this case, e.g. activities occurring in groups of neurons located deep in the brain or occurring in asymmetrically distributed neurons.^8^ Consequently, the neo-cortex emerges as the main structure involved in the generation of ERPs. Approximately 70% of its neuron network is composed of pyramidal cells in which the dendrites extend from more deeply located somas towards more superficial cortical layers.^9^

Recording and analyzing ERPs is currently one of the most useful procedures employed by cognitive neuroscience researchers to investigate the process of retrieval.^10^ Its capacity to directly demonstrate the electrophysiological brain activity elicited by discrete experimental events makes this procedure a major approach in this field, together with fMRI and PET techniques. Although a technique called magnetoencephalography (MEG) also provides accurate time-course data and is slightly more precise regarding spatial resolution than ERP,^11^ its instrumentation costs are extremely high compared with ERP, representing a major limitation to its implementation.^12^

## ERP and memory retrieval

The process of retrieval may be divided into three main stages:^1^ pre-retrieval processes, when an external stimulus (cue) is initially processed;^2^ retrieval process (also known as an ecphoric moment), when the interaction between an external stimulus and a memory trace is held; and^3^ post-retrieval processes, when the retrieved memory is monitored and evaluated.^13,14^ Below, we review the major literature concerning each retrieval stage.

## Pre-retrieval processes

Memory retrieval processes usually involve interaction between an external cue and a specific memory representation or memory trace.^14^ The quality of the cue-trace interaction and consequently its capacity to retrieve a memory successfully, vary according to the way in which the external cues are initially processed.^13^ According to some authors,^15^ the possibility of varying the way a cue is initially processed depends on the adoption of a specific “retrieval orientation”, which may be defined as a pre-retrieval cognitive state that influences the cue processing in order to facilitate the retrieval of a specific type of memory trace. In other words, the retrieval orientation determines the particular form of processing that should be employed for the recovery of a particular type of information.^16^ For example, individuals adopt a different retrieval orientation according to whether a memory test requires the retrieval of pictorial or verbal information. The neural activities elicited by this kind of processing are revealed when research subjects are required to remember different types of stored information (e.g. pictures versus words).^17^

This operation is essential for the recollection process. When individuals attempt to remember a specific episode, enormous quantities of interfering episodes and environmental information must be rejected in order to recover the desired information. This suggests that remembering also relies on a variety of control and planning mechanisms held before the retrieval of a memory trace. The pre-retrieval process, called retrieval orientation, is also responsible for determining the specific type of processing that should be applied to a retrieval cue in order to make a given memory retrieval possible, avoiding the interference of undesired information.^15^

The first ERP research which clearly revealed this process^18^ found that people employed different retrieval orientations while attempting to remember ‘shallow’ versus ‘deeply’ encoded words. The words presented after the shallow encoding elicited waveforms with more positive voltages than the deeply encoded words. This voltage difference was maximal over central and frontal scalp regions between 200 and 600 ms post-stimulus onset. The employment of semantic versus phonological stimuli^19^ revealed that a certain level of difficulty is needed to induce the expected retrieval orientation ERP effects. These results were challenged later^20^ by the demonstration that a remarkable ERP difference according to the nature of the target material (words/pictures) was present, regardless of difficulty. The ERP effect elicited when participants attempted to remember pictures instead of words consisted of topographically widespread electrical negativity with an onset of around 250 ms post-stimulus and sustained for more than 1000 ms.^16,20^

The utilization of pictures versus words as stimuli to study orientation retrieval was present in some subsequent experiments,^17^ which demonstrated that the magnitude of the ERP effect elicited by orientation retrieval is proportional to the difference between the encoded stimuli and the external cues involved in the retrieval task, suggesting that these effects might reflect the cognitive processing necessary to improve the overlap between external stimuli and internal memory traces. A subsequent study^21^ investigating direct (yes/no) versus indirect (semantic judgment) tests verified that when direct tests were used, a replication of previous findings was evident,^17^ but when indirect tests were employed, a much weaker ERP effect was induced, leading the authors to conclude that ERP correlates of retrieval orientation are restricted to direct tests, corresponding exclusively to explicit episodic retrieval operations.

Further studies^22^ demonstrated that ERP effects elicited by a picture/word exclusion tasks,^23^ onset earlier and offset later, suggesting that participants adopt different retrieval orientations strategies according to the demands of the retrieval task. These authors also demonstrated in a later study that distinct retrieval orientation ERP effects elicited by words and pictures become absent when subjects are instructed to switch their strategy on a trial basis. They concluded that retrieval orientation tends to be a tonically sustained activity and the adoption of a given retrieval orientation strategy develops over multiple trials rather than on a trial by trial basis.^24^

Two further studies approached the interactions of retrieval orientation with other memory concepts. The first^25^ suggested that the ERP correlates of ‘heuristic distinctiveness’, a response mode in which participants expect to make the recognition decision based on vivid details of objects, are similar to the ERP correlates of retrieval orientation. Regarding possible interactions between retrieval orientation and conceptual versus perceptual memory, the second study^26^ showed that there are specific ERP signatures related to the retrieval orientation for each of these types of memory.

## Retrieval processes

Currently, there are two main theoretical perspectives which seek to explain the process of retrieval. The perspective of the single process models, which conceives recognition as a one-dimensional scalar value of memory strength;^27^ and the perspective of the dual process models, which posits that recognition is served by two independent processes: recollection and familiarity.^28^ Akin to the single process perspective, the dual process models also suggest that recognition judgments are supported by a strength-like type of information, referred to by this perspective as familiarity. However, this perspective suggests that recognition is also supported by recollection, a second and functionally distinct type of recognition memory which involves the retrieval of qualitative information from the encoded episode.^10^ A useful example of familiarity is the situation in which we meet a person and even though we might be confident that we know this person, there is no retrieval of any specific information, such as their name or where we have met previously. An example of recollection, on the other hand, is when we meet a person and more detailed information is remembered such as the name, professional position or place of abode of the person.

Even though dual process models remain controversial, they have been increasingly supported by behavioral,^29^ animal,^30^ amnesic patients,^31^ functional magnetic resonance imaging (fMRI),^32^ and ERP findings.^10^ ERPs, which provide the timing and scalp distribution of brain processes that are regularly associated with stimulus processing,^33^ can be employed as a powerful tool to doubly dissociate the recollection and familiarity processes, as well as to examine the manifestations of each process separately.

Initial ERP studies aiming to explore electrophysiological correlates of retrieval, employed old-new recognition tasks, that is, they contrasted the activity induced by correctly classified old versus correctly classified new items. The studies employing this approach demonstrated different ERP activity induced by these distinct conditions,^13^ consisting in a more positive polarity for old versus new items. Considering the dual process model however, the simple contrast between old and new items was proven problematic, since it does not discriminate recollection from familiarity. Alternative experimental designs were employed to address this limitation. These designs entailed instructing participants to allocate experimental items as ‘Remembered’ versus ‘Known’;^34-40^ contrasting accurate versus inaccurate source memory judgments;^41,42^ associative versus item recognition;^43^ deep versus shallow encoding;^18^ and contrasting recognition of old items versus false alarms.^44-45^

The ERP studies based on the designs cited above demonstrated that two major effects are produced during the recognition task. The positive-going and predominantly frontal effect, called the ‘mid-frontal old/new effect’, which onsets around 300–400 ms post-stimulus onset and is thought to reflect familiarity, and a positive-going effect over the parietal sites called ‘left parietal old/new effect’, which onsets around 400–500 ms post-stimulus, frequently more prominent over the left hemisphere and is assumed to be a correlate of recollection.^10^ The functional significance of the mid-frontal effect remains uncertain. Although some authors hold the view that it simply represents priming rather than familiarity, this remains a question for future research. The left parietal effect, as supported by fMRI findings,^46^ reflects the involvement of the parietal cortex in attentional activity related to recollection or the neural activity that could contribute causally to recollection-based memory judgments.^13^

## Post-retrieval processes

The process of retrieval is assumed to be followed by post-retrieval operations attempting to monitor and evaluate the information recovered. Post-retrieval processes are important to manipulate the products of retrieval according to current behavioral goals (e.g. keep the representation of a studied episode in the consciousness to make a decision). There is evidence that right frontal areas of the brain could be involved in this process. More specifically, as demonstrated by fMRI data, the right dorsolateral prefrontal cortex is at least partially involved in this process.^13^

ERP research has corroborated the involvement of these regions in post-retrieval activity. The ‘right frontal old/new effect” is a positive ERP effect (old items eliciting a more positive voltage than new items) reaching a peak over the right frontal electrodes.^41^ This effect onsets at around 800 ms and is often sustained for more than one second, suggesting that it reflects a relatively slow process. This is congruent with the idea that post-retrieval processes are slower, onsetting later than pre-retrieval or retrieval operations. This effect was demonstrated by studies employing a variety of memory retrieval tasks.^18,40-42,47-51^

Currently, the view that the right frontal effect is a correlate of post-retrieval processes has been challenged by two fMRI studies.^52,53^ Their findings suggested that the right frontal activity is primarily a correlate of decision-making operations held during the retrieval task. A current ERP study corroborated this data by demonstrating that this effect was also elicited by semantic judgments, even when the participants did not retrieve the previously learned stimuli,^54^ that is, this effect was also elicited when participants were instructed to make a semantic judgment with objects shown for the first time in the test (new objects).

## Critical analysis

This review of the literature regarding memory retrieval and ERP revealed some important issues for debate. Initially in this session we shall criticize the theoretical assumptions generated by the ERP research concerning each memory retrieval process. The study then addresses more instrumental problems, including what ERP can actually offer over and above alternative procedures, and the feasibility of conducting ERP research in national research centers.

Considering retrieval orientation, the data reviewed above demonstrated consistent differences in brain activity according to type of stimuli retrieved. Moreover, these activity differences were not material-specific, i.e., findings were replicated in experiments employing a variety of material as stimuli.^19,20^ These ERP differences were mostly measured around 200–250 milliseconds post-stimulus onset and present at most electrodes,^18^ corroborating the idea that this reflects fast pre-retrieval operations. These ERP differences were modulated by cognitive demands,^22^ difficulty level,^17^ and were induced mainly by direct memory tests.^21^

Perhaps the most important data generated by retrieval orientation studies is the time-frame within which the pre-retrieval process occurs. Since pre-retrieval operations are assumed to take place before retrieval, it is a remarkably fast process. By employing ERPs, it was possible to reveal the retrieval orientation brain activity in short time-frames that cannot be investigated by any other cognitive neuroscience instrument. Although some retrieval orientation studies employing fMRI were later conducted,^21,55^ they drew on previous ERP findings. Without the above-mentioned retrieval orientation ERP studies, it would be difficult to show the timing or even the existence of such an early process.

As mentioned previously, a current issue in retrieval memory research is the debate concerning single versus dual process models.^27,56^ Single process models of recognition have been the most commonly employed theoretical approach to recognition in the last decades,^27^ and although the model remains widely used, increasing evidence has demonstrated that recollection and familiarity might consist of functionally and behaviorally different processes,^35^ lending credence to dual process models.

The debate concerning these two models does not seem to be nearing consensus. ERP research seeking to dissociate recollection and familiarity have been providing evidence in favor of dual process models, demonstrating distinct ERP correlates of recollection (parietal old/new effect) and familiarity (midfrontal old/new effect).^10,33,57^, Despite the time-course and topographical differences between these models, a strong double dissociation between them has not yet demonstrated. The reason for this lies in the experimental difficulty of getting participants to perform recollection-based recognition, without them also engaging in familiarity-based processing.^57^ It is a challenging issue to be pursued by future research.

After the retrieval of stored information, evaluative and monitoring operations take place which have been linked to a later frontal ERP effect elicited at around 800 ms poststimulus.^18^ New and compelling evidence has suggested that this effect is probably a result of decision-making processes required in most recognition memory tasks (e.g. deciding whether an object is old or new).^54^

While more research is needed to identify which cognitive processes are indeed correlated with this late ERP effect, this debate exemplifies the interplay between ERP and fMRI data towards the investigation of a specific issue. In this case, the ERP data^54^ supports the fMRI data,^52,53^ adding further information to the latter, showing for example, that the allegedly frontal brain correlate of decision-making processing onsets at around 800 ms post-stimulus.

It is important to note that the three stages of memory retrieval outlined above were, to a large extent, initially demonstrated by ERP data.^13^ The ERP findings demonstrated a time-line ranging from a more initial cue processing stage, named pre-retrieval (evident after 200 ms), including the retrieval processing itself, between 300–500 ms, and processes maintained after retrieval, beginning at around 800 ms and often lasting for 1000 ms. These data show the importance of an instrument capable of measuring brain activations with such time precision. This is the reason why it has also been employed in the investigation of a variety of cognitive processes in which time is an important issue, such as language,^58^ perception,^59^ priming,^60^ etc.

Despite its timing advantages, a limitation of the ERP technique is its lack of spatial precision, i.e., electrical activities elicited in deep locations in the brain as well as in asymmetrically distributed neurons, are not able to be recorded at the scalp.^8^ Even electrical activity generated in the neocortex is not recorded with the same precision as the fMRI data (see 61 for an example of advances in this approach).

These characteristics of the data provided by ERPs, namely, time precision and spatial limitation, raises questions about what is actually important when correlating brain activity with cognition. A possible answer for this may involve further questions, such as which cognitive process is one aiming to study? Which aspects of a given process are intended to be investigated? Is the goal to reveal the time-course of a given cognitive process or to point out specific brain areas involved in it?

In the present article the advantages of ERP in the study of memory retrieval are clear. Its capability to establish the time-course of different ‘sub-operations’ involved in the process of retrieval is not achievable by alternative imaging techniques, exemplifying the unique and important data this approach can generate.

It is also true that the fMRI data provided by earlier studies have made important contributions toward the understanding of retrieval, and often, information that can be provided only by this technique.^46^ Therefore, an important approach to this time/space problem is the attempt to integrate the data generated by both sources. Clearly, due to electromagnetic reasons, it is not possible to use both techniques simultaneously in the same subject, but the same experimental paradigm may be adopted for both techniques.^62,63^ This association may represent a further advance in the understanding of brain and cognition, providing more accurate data concerning timing and structural activity.

Research in cognitive neuroscience is often an expensive investment, because it usually involves the acquisition of cutting-edge technology to enable scientists to observe the brain activity related to cognitive activity. The funds needed to acquire an fMRI scanner for example is up to one million U.S. Dollars. It is also expensive to conduct experiments, given each scanning hour may cost between 400–600 U.S. Dollars. The acquisition and usage costs of the ERP apparatus, on the other hand, are much lower. A thirty-three channel apparatus, for example, costs little more than twenty to thirty thousand U.S. Dollars, and there is no further extra costs to conduct experiments.

Considering the important data ERP devices provide, they seem a viable alternative for Brazilian laboratories. Currently, there are only a few laboratories in Brazil employing ERP in their research, and even less applying it within the modern cognitive neuroscience framework (see 58 for an outstanding example). This might be explained by the lack of scientists with the appropriate skills to conduct such experiments, where a possible solution might be to forge links with foreign researchers who conduct this kind of study.

As a final point, it is important to highlight that although ERPs have already been used in research for some four decades,^64^ they remain routinely used world wide. New advances in data analysis^5^ and electrode montages^2,61^ have been made. Its timing advantage makes this low-cost technique an exceptional way to explore brain-cognition interaction in humans. It represents an important route towards a broader conception of the human mind as well as its brain representations.
